# Evolutionary History of *Triticum petropavlovskyi* Udacz. et Migusch. Inferred from the Sequences of the 3-Phosphoglycerate Kinase Gene

**DOI:** 10.1371/journal.pone.0071139

**Published:** 2013-08-19

**Authors:** Qian Chen, Hou-Yang Kang, Xing Fan, Yi Wang, Li-Na Sha, Hai-Qin Zhang, Mei-Yu Zhong, Li-Li Xu, Jian Zeng, Rui-Wu Yang, Li Zhang, Chun-Bang Ding, Yong-Hong Zhou

**Affiliations:** 1 Triticeae Research Institute, Sichuan Agricultural University, Sichuan, People's Republic of China; 2 Key Laboratory of Crop Genetic Resources and Improvement, Ministry of Education, Sichuan Agricultural University, Sichuan, People's Republic of China; 3 College of Resources and Environment, Sichuan Agricultural University, Sichuan, People's Republic of China; 4 College of Biology and Science, Sichuan Agricultural University, Sichuan, People's Republic of China; Nanjing Agricultural University, China

## Abstract

Single- and low-copy genes are less likely to be subject to concerted evolution. Thus, they are appropriate tools to study the origin and evolution of polyploidy plant taxa. The plastid 3-phosphoglycerate kinase gene (*Pgk-1*) sequences from 44 accessions of *Triticum* and *Aegilops*, representing diploid, tetraploid, and hexaploid wheats, were used to estimate the origin of *Triticum petropavlovskyi*. Our phylogenetic analysis was carried out on exon+intron, exon and intron sequences, using maximum likelihood, Bayesian inference and haplotype networking. We found the D genome sequences of *Pgk-1* genes from *T. petropavlovskyi* are similar to the D genome orthologs in *T. aestivum*, while their relationship with *Ae. tauschii* is more distant. The A genome sequences of *T. petropavlovskyi* group with those of *T. polonicum*, but its *Pgk-1* B genome sequences to some extent diverge from those of other species of *Triticum*. Our data do not support for the origin of *T. petropavlovskyi* either as an independent allopolyploidization event between *Ae. tauschii* and *T. polonicum*, or as a monomendelian mutation in *T. aestivum*. We suggest that *T. petropavlovskyi* originated via spontaneous introgression from *T. polonicum* into *T. aestivum*. The dating of this introgression indicates an age of 0.78 million years; a further mutation event concerning the B genome occurred 0.69 million years ago.

## Introduction

In the Xinjiang region of China, *Triticum* species are abundant. The Xinjiang rice wheat (*Triticum petropavlovskyi* Udacz. et Migusch.), known as ‘Daosuimai’ or rice-head wheat, is one of the Chinese endemic wheat landraces, together with the Sichuan white wheat complex (*T. aestivum* L.), Tibetan weedrace (*T. aestivum* ssp. *tibetanum* Shao) and Yunnan hulled wheat (*T. aestivum* ssp. *yunnanense* King) [1]. Based on chromosome pairing, morphology, eco-geographical origin and RFLP analysis, the Xinjiang rice wheat is distinct from other Chinese endemic wheat landraces [2]–[5].

The origin of *T. petropavlovskyi* has been discussed for decades. Gorsky [6] analyzed the morphology of Xinjiang rice wheat, and suggested that it was a mutated form of the tetraploid *Triticum polonicum* L.. However, Udachin and Miguschova [7] discovered that the Xinjiang rice wheat is not tetraploid but hexaploid, and named it *T. petropavlovskyi* Udacz. et Migusch. The chromosomal constitutions of the Xinjiang rice wheat is AABBDD [2], [8]–[10]. Dorofeev et al. [11] hypothesized that *T. petropavlovskyi* could be the result of spontaneous hybridization between *T. aestivum* and *T. polonicum*. Several previous studies indicated that the genes supporting a long glume in *T. polonicum* and *T. petropavlovskyi* were allelic and located on the long arm of chromosome 7A [12]–[14], agreeing with the hypothesis of spontaneous hybridization. Yen et al. [15] studied the natural distribution in Xinjiang of *Aegilops tauschii*, and Yang et al. [16] and Liu et al. [17] reported a similar distribution for a dwarfing accession of *T. polonicum* and suggested that *T. petropavlovskyi* is derived from a hybridization event between *T. polonicum* and *Ae. tauschii*. However, Efremova et al. [18] maintained that *T. petropavlovskyi* originated from *T. aestivum* through spontaneous mutation.

Despite prior intensive research, the origin of *T. petropavlovskyi* is still uncertain, and three hypotheses have been proposed: (1) *T. petropavlovskyi* is an independent species is derived from a natural hybridization event between *T. polonicum* and *Ae. tauschii* [10], [15], [19]–[21]; (2) *T. petropavlovskyi* is a natural cross or backcross between *T. polonicum* and *T. aestivum* [2], [11], [12], [22], [23]; and (3) *T. petropavlovskyi* is a monogenic mutant of *T. aestivum* [18], [24]. In a recently study, Kang et al. [25] created the synthetic hexaploid wheat (SHW-DPW) between *T. polonicum* from Xinjiang and *Ae. tauschii*: its spike morphology was similar to *T. petropavlovskyi*. However, a comparison of SHW-DPW to *T. petropavlovskyi*, *T. polonicum* and related species by the phylogenetic analysis of the *Acc-1* gene indicated that *T. petropavlovskyi* originated from the cross between *T. polonicum* from Xinjiang and exotic landraces of *T. aestivum* [26]. This finding contradicts the morphology-based conclusion of preceding study. Previous works based on different methods, including cytology, morphology and nuclear markers, have failed in identifying the origin of *T. petropavlovskyi*. Furthermore, no molecular-clock has been reported to examine the timing of its origin.

Single- and low-copy nuclear genes, being less susceptible to concerted evolution [27]–[29], are useful in phylogenetic study [30]–[33] as well as in the identification of parents of allopolyploidy taxa [26], [34]–[36]. Genes such as acetyl-CoA carboxylase 1 (*Acc-1*) [30], disrupted meiotic cDNA 1 (*DMC1*) and translation elongation factor G *(EF-G)* [37] have been particularly useful in elucidating the phylogenesis of *Triticum-Aegilops* species. The plastid 3-phosphoglycerate kinase (PGK) gene, *Pgk-1*, is a single copy nuclear gene in diploid species of the Triticeae; it is frequently considered to be superior to the *Acc-1* gene for assessing the evolutionary history of polyploid wheats, because the *Pgk-1* gene has more parsimony informative sites than the *Acc-1* gene [31], [38]. For allotetraploid and allohexaploid species with two or three copies of genes present as single copies in diploid ancestors, the *Pgk-1* gene can both elucidate the phylogenetic relationships of such polyploid as well as potential progenitors [30], [31], [39], [40].

In this study, we sequenced and analyzed the single-copy nuclear *Pgk-1* gene in the following taxa: *T. petropavlovskyi*, SHW-DPW (synthetic hexaploid wheat between *T. polonicum* and *Ae. tauschii*), and the hypothetical *Triticum* and *Aegilops* progenitors of *T. petropavlovskyi* to reveal their phylogenetic relationships and to explore both the origin of *T. petropavlovskyi* and its divergence time from other taxa.

## Materials and Methods

### Plant materials

The species, genomic constitutions, origin, GenBank accessions, and sources of the taxa are listed in Table 1. The sequences of *pgk-1* gene of the accensions with TA numbers were obtained from the GenBank database; the rest of the species considered for the sequences are reported here for the first time.

**Table 1:** Plants used in this study.

Species	Genome	Accession	Origin	Abbrev.	GenBank Ac. No.
*Triticum urartu* Thum. ex Gandil.	A^u^	TA763	Lebanon	TUR63A	AF343474
*Aegilops bicornis* (Forskal) Jaub. et Spach.	S^b^	TA1954	Egypt	AEB954S	AF343485
*Aegilops longissima* Schweinf. et Muschl.	S^l^	TA1912	Israel	AEL912S	AF343487
*Aegilops searsii* Feldman et Kislev	S^s^	TA2355	Israel	AES355S	AF343489
*Aegilops sharonensis* Eig	S^sh^	TA2065	Turkey	AES065S	AF343486
*Aegilops speltoides* Tausch	S	TA2368	Turkey	AES368S	AF343483
*Aegilops speltoides* var. *ligustica* (Savign.) Fiori	S	TA1770	Iraq	AEL770S	AF343484
*Aegilops tauschii* Cosson	D	AS60	Middle East	AET60D	JQ327050
		TA1691	Unkown	AET691D	AF343479
*Triticum polonicum* L.	AB	AS302	Xinjiang, China	TPO302A	JQ327101
				TPO302B	JQ327102
		AS304	Xinjiang, China	TPO304A	JQ327088
				TPO304B	JQ327089
		PI42209	Australia	TPO209A	JQ327096
				TPO209B	JQ327097
*Triticum turgidum* L.	AB	AS2233	Xinjiang, China	TUR233A	JQ327113
				TUR233B	JQ327114
		AS2277	Xinjiang, China	TUR277A	JQ327077
				TUR277B	JQ327078
*Triticum durum* Desf.	AB	AS2349	Xinjiang, China	TDU349A	JQ327115
				TDU349B	JQ327116
*Triticum durum* Desf. cv. Langdon	AB	LDN	USA	TDULA	JQ327057
				TDULB	JQ327058
*Triticum turanicum* Jakubz.	AB	AS2229	Xinjiang, China	TTU229A	JQ327109
				TTU229B	JQ327110
		AS2279	Xinjiang, China	TTU279A	JQ327111
				TTU279B	JQ327112
*Triticum dicoccoides* (Koern. ex Aschers. et Graeb.) Schweinf.	AB	TA51	Israel	TDI51A	AF343481
				TDI51B	AF343476
		AS838	Xinjiang, China	TDI838A	JQ327075
				TDI838B	JQ327076
*Triticum carthlicum* Nevski (syn. *T. persicum* Vav.)	AB	PI532494	Kars, Turkey	TCA494A	JQ327065
				TCA494B	JQ327066
		PI532509	Xinjiang, China	TCA509A	JQ327073
				TCA509B	JQ327074
*Triticum timopheevii* (Zhuk.) Zhuk.	AG	TA2	Armenia	TTI2A	AF343477
				TTI2G	AF343488
		PI94761	Georgia, USA	TTI761A	JQ327126
				TTI761G	JQ327127
*Triticum petropavlovskyi* Udacz. et Migusch.	ABD	AS358	Xinjiang, China	TPE358A	JQ327090
				TPE358B	JQ327091
				TPE358D	JQ327092
		AS359	Xinjiang, China	TPE359A	JQ327103
				TPE359B	JQ327104
				TPE359D	JQ327105
		AS360	Xinjiang, China	TPE360A	JQ327106
				TPE360B	JQ327107
				TPE360D	JQ327108
*Triticum aestivum* L. ssp. *tibetanum* Shao	ABD	AS1026	Xizang, China	TTB1026A	JQ327123
				TTB1026B	JQ327124
				TTB1026D	JQ327125
		AS1027	Xizang, China	TTB1027A	JQ327062
				TTB1027B	JQ327063
				TTB1027D	JQ327064
*Triticum aestivum* L. ssp. *yunnanense* King	ABD	AS331	Yunnan, China	TYU331A	JQ327131
				TYU331B	JQ327132
				TYU331D	JQ327133
		AS338	Yunnan, China	TYU338A	JQ327085
				TYU338B	JQ327086
				TYU338D	JQ327087
		AS343	Yunnan, China	TYU343A	JQ327128
				TYU343B	JQ327129
				TYU343D	JQ327130
*Triticum sphaerococcum* Perciv.	ABD	PI70711	Iraq	TSP711A	JQ327117
				TSP711B	JQ327118
				TSP711D	JQ327119
		PI115818	Punjab, India	TSP818A	JQ327093
				TSP818B	JQ327094
				TSP818D	JQ327095
*Triticum macha* Dekapr. et Menabde.	ABD	PI278660	UK	TMA660A	JQ327082
				TMA660B	JQ327083
				TMA660D	JQ327084
*Triticum spelta* L.	ABD	PI347852	Switzerland	TPL852A	JQ327098
				TPL852B	JQ327099
				TPL852D	JQ327100
		PI347858	Switzerland	TPL858A	JQ327120
				TPL858B	JQ327121
				TPL858D	JQ327122
*Triticum compactum* Host	ABD	PI124298	Unknown	TCO298A	JQ327070
				TCO298B	JQ327071
				TCO298D	JQ327072
		PI352299	Switzerland	TCO299A	JQ327067
				TCO299B	JQ327068
				TCO299D	JQ327069
*Triticum aestivum* L. cv. Chinese Spring	ABD	CS	Sichuan, China	TCHSA	JQ327051
				TCHSB	JQ327052
				TCHSD	JQ327053
*Triticum aestivum* L. cv. Chuannong-16	ABD	CN16	Sichuan, China	TCN16A	JQ327054
				TCN16B	JQ327055
				TCN16D	JQ327056
*Triticum aestivum* L. cv. J-11	ABD	J-11	Sichuan, China	TJ11A	JQ327079
				TJ11B	JQ327080
				TJ11D	JQ327081
Synthetic hexaploid wheat	ABD	SHW-DPW		SHWDA	JQ327059
				SHWDB	JQ327060
				SHWDD	JQ327061
*Psathyrostachys juncea* (Fischer) Nevski	Ns	PI222050	Afghanistan	PJU050N	FJ711031

The Genebank with AF numbers are from Huang et al. [25], those with JQ numbers have been assinged in this study.

The accessions with PI and AS numbers were kindly provided by the American National Plant Germplasm System (Pullman, Washington, USA) and the Triticeae Research Institute, Sichuan Agricultural University, China, respectively. The artificial synthetic amphiploid of *Triticum polonicum* and *Aegilops tauschii* (SHW-DPW) was produced by Kang et al. [25]. The plants and voucher specimens have been deposited at Herbarium of Triticeae Research Institute, Sichuan Agricultural University, China (SAUTI).

### DNA extraction, amplification and sequencing

DNA was extracted from fresh leaves of single plants, following a standard CTAB (cetyltrimethylammonium bromide) protocol [41]. For amplification of the *Pgk-1* gene, a pair of *Pgk1*-specific primers, PPF1 (5′-CACCTGGGTCGTCCTAAGGGTGTT-3′) and PPR1 (5′-ACCACCAGTTGTGTTGTGGCTCAT-3′), was used [31]. Polymerase chain reactions (PCR) were performed in a GeneAmp 9700 Thermal Cycler (Applied Biosystems Inc., California, USA) according to the following cycling program: initial denaturation at 94°C for 5 min; 35 cycles of 94°C for 30 s, 56°C for 30 s, 68°C for 5 min; followed by a final elongation period at 68°C for 10 min. A final volume of 50 µl for each PCR reaction was prepared, containing 0.5 µg of genomic DNA, 10× reaction buffer, 1.5 mM of each primer, 2.5 mM of each dNTP, 2.5 mM MgCl2, 2 units of high-fidelity Ex*Taq* DNA polymerase (Takara Biotechnology Co. Ltd., Dalian, China).

1.0% agarose gel was used to estimate the size of the amplification products, which were purified using the EZNATN gel extraction kit (Omega Bio-Tech, Georgia, USA) and stored in 30 µl TE buffer. The purified products were cloned into the pMD19-T vector (Takara) according to the manufacturer's instructions. Cloning of PCR amplifications from single-copy nuclear genes from allopolyploid species should isolate homoeologous sequences from each nuclear genome [42], [43]. For the hexaploid *Triticum* species, the A, B and D genomes homoeologous sequences of *Pgk-1* gene were isolated, and the A and B genomes homoeologous sequences were separated for the tetraploid *Triticum*. The cloned PCR products were commercially sequenced on both strands by the Beijing Genomics Institute (BGI, Shenzhen, China). All the sequences used in the phylogenetic analysis were derived from at least five independent clones.

### Alignments and phylogenetic analysis

Multiple sequences were aligned using Clustal X with default parameters, followed by manual adjustment to minimize gaps [44]. In an initial phylogenetic analysis, if all sequences used for alignment derived from independent clones, formed a monophyletic group, then only one sequence was used later on. Distinct sequences derived from single accessions mapping different clades were all included in the phylogenetic analysis. Nucleotide frequencies, transition/transversion ration, and variability in different regions of the sequences were examined by MEGA 5.0 [45].

Three data matrixes, including exon+intron data (the target *Pgk-1* gene sequences), exon data and intron data, were used separately to implement phylogenetic analyses. Phylogenetic trees were created using Maximum likelihood (ML) and Bayesian inference (BI). ML analysis was carried out with PAUP*4.0b10 (Swofford, D. L., Sinauer Associates, http://www.sinauer.com), and *Psathyrostachys juncea* (Fischer) Nevski was used as an outgroup. The best-fit models of sequence evolution for ML analysis were estimated using ModelTest v3.0 with Akaike information criteria (AIC) [46]. The optimal models were found to be HKY+G for the exon+intron data, TVM+G for the intron data, and TrN+G for exon data. ML heuristic searches were performed with 100 random addition sequence replications and TBR branch swapping algorithm. The robustness of the trees was estimated using bootstrap support (BS) [47]. ML bootstrapping was performed with 250 replicates, each with three replicates of stepwise random taxon addition, using the same model and parameters. BS-values under 50% were not included in figures.

Bayesian inference (BI) analysis was performed using MrBayes v3.2 [48] with *Psathyrostachys juncea* used as an outgroup. The best-fit models for BI analysis were carried out with AIC using MrModelTest v2.3 (http://www.ebc.uu.se/systzoo/staff/nylander.html). The optimal models were found to be GTR+I+G for exon+intron data, GTR+G for intron data and exon data. Four MCMC (Markov Chain Monte Carlo) chains (one cold and three heated) were applied with default setting. In order to make the standard deviation of split frequencies fall below 0.01, 4,200,000 generations for exon+intron data, 3,000,000 generations for intron data, and 5,000,000 for exon data were run. Samples were taken every 100 generations under the best-fit model. For all analyses, the first 25% of samples from each run were discarded as “burn-in” to ensure the stationarity of the chains. Bayesian posterior probability (PP) values were obtained from a majority rule consensus tree generated from the remaining sampled trees. PP-values less than 90% were not included in figures.

### Network analysis

The median-joining (MJ) network method [49], and Templeton, Crandall and Sing (TCS) method [36], have been shown to be effective methods for revealing specific progenitor descendant relationships of perennial Triticeae [21], [50]–[52], and were thus performed in this study. Before reconstructing the MJ and TCS networks, a test of recombination was performed using the Phi (Pairwiser homoplasy index) method within Splits tree [53]. Building upon this test, the sequences of A (P = 0.64932), B (P = 0.9999) and D genomes (P = 1.0) were used to generate the MJ network, and the exon data (P = 0.7493) was used to generate the TCS network. MJ network analysis was generated by Network 4.1.1.2 program (Fluxus Technology Ltd, Clare, Suffolk, UK), and TCS haplotype network was performed to evaluate possible genetic relationships between haplotypes with the computer program TCS 1.2.1 [54].

### Divergence dating

The potential clock-like evolution of *Pgk-1* sequences was evaluated with a likelihood ratio rate comparing the likelihood scores from the unconstrained and clock-constrained analyses, implemented in PAUP*4.0b10. The molecular clock was rejected because the substitution rates were significantly heterogeneous (χ2 = 154.90, df = 95, P = 0.0001), implying a very poor fit to the molecular clock. Therefore, divergence times with 95% confidence intervals (C.I.) were estimated using the Bayesian relaxed molecular clock method, implemented in BEAST v1.7.1 [55].

The lack of fossils for Triticeae precluded a direct calibration of tree topologies. Instead, molecular dating of the intron data was estimated on the basis of the intron region of the *Pgk-1* gene clock of 0.0051 substitutions per site per MY (million year) [31], [38], setting the clock for the divergence of Pooideae and Panicoideae sub-families at 60 MYA [56]–[58]. Calibration points were performed using a relaxed uncorrelated lognormal molecular clock. MCMC searches were run for 10,000,000 generations under GTR+G model (with the associated parameters specified by MrModelTest as the priors), with the first 2,000,000 discarded as burn-in. Trees were then viewed in FigTree v1.3.1 (http://tree.bio.ed.ac.uk/).

## Results

### Sequence analysis

**Figure 1:**
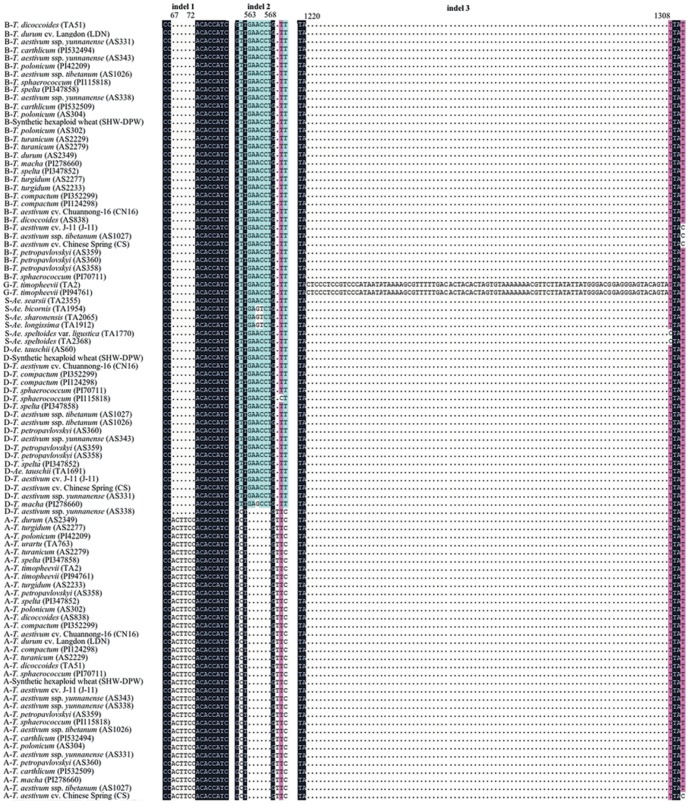
Maximum-likelihood tree from the exon+intron sequences of the *Pgk-1* gene of *T. petropavlovskyi* and its related species. Numbers above nodes are bootstrp values >50% numbers below nodes are posterior probability values >90%. Genome composition, species name and accession number/cultivar name are indicated for each taxon.

In all polyploid species considered, the expected number of copies of the *Pgk-1* gene were successfully amplified. The DNA sequence of the *Pgk-1* gene includes 5 exons and 4 introns, which range in length from 1360 bp to 1476 bp, as known from previous study [30], [31], [38] (Table 2). The lengths of exon+intron, exon and intron data sets were 1466, 894 and 572 bp, respectively. As expected, the level of nucleotide variation in the exon region (88 variable sites and 36 parsimony-informative sites) was lower than that in the intron region (107 variable sites and 80 parsimony-informative sites). The average content of G+C of exon+intron, and exon was 42.9, and 47.3%, respectively, and the transition/transversion ratio was 2.13, and 2.24, respectively. The alignment of the exon sequence was unambiguous and without gaps. Gaps were, on the contrary, present in introns. In particular, apart from single nucleotide substitutions and deletions, three significant indels (insertion/deletion) (indel 1, 2, 3) were found (Fig. 1). Indel 1 was located at position 67–72 of the A genome; indel 2 mapped at position 563–568 and had a 6 bp deletion specific for A genome. Unexpectedly, the *T. aestivum* ssp. *yunnanense* (AS338) did not show the indel 2. Indel 3 was presented at position 1220–1308 and had a 89 bp insertion in the G genome of *T. timopheevii*.

**Table 2:** Parameters derived from *Pgk-1* sequence alignements.

	Total sites	Variable characters	Conversed characters	Informative characters
Exon+Intron	1466	174	1292	91
Exon	894	88	806	36
Intron	572	107	378	80

### Phylogenetic analyses

**Figure 2:**
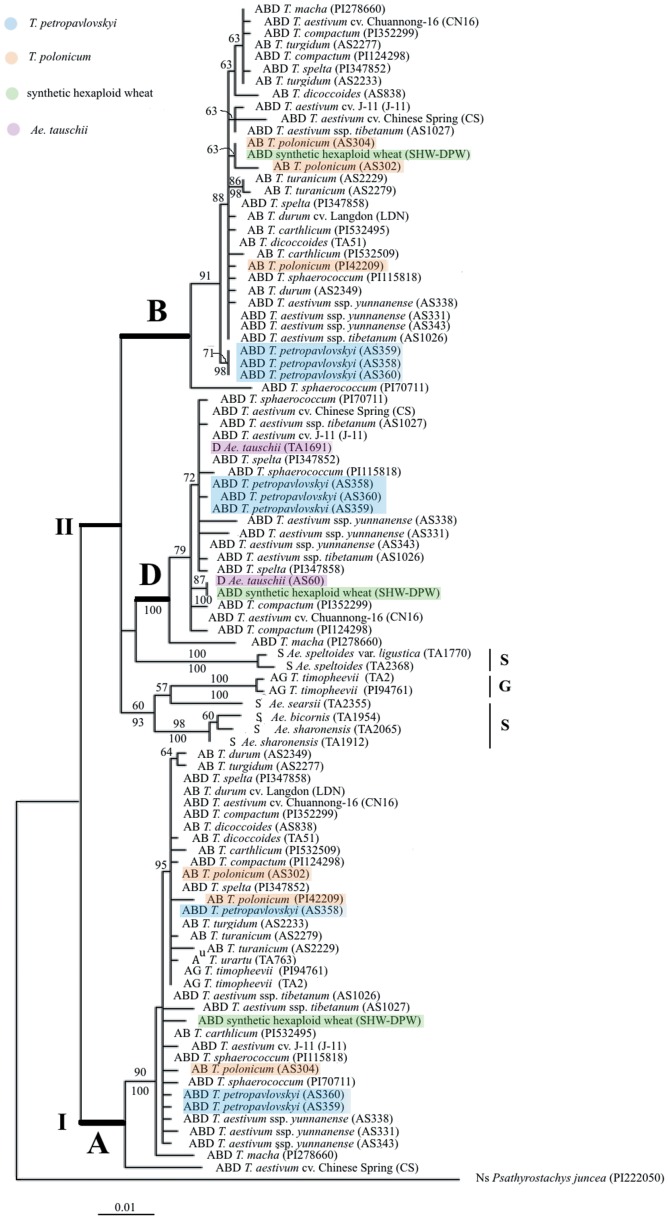
Maximum-likelihood tree from the exon+intron sequences of the *Pgk-1* gene of *T. petropavlovskyi* and its related species. Numbers above nodes are bootstrp values ≥50% numbers below nodes are posterior probability values ≥90%. Genome composition, species name and accession number/cultivar name are indicated for each taxon.

Using *Psathyrostachys juncea* as an outgroup, the three data sets corresponding to exon+intron, exon and intron were used phylogenetic analyses (ML and BI) were carried out. ML analysis of the exon+intron data generated a single phylogenetic tree (−Lnlikelihood = 4092.85), with the following parameters: A = 0.26; C = 0.21; G = 0.23, T = 0.30, gamma shape parameter = 0.29. Bayesian analysis of the same data recovered the same topology. In Figure 2, the ML tree is reported with values of the bootstrap support (BS) above and posterior probabilities (PP) below branches.

The ML tree of Figure 2 indicates that all homoeologous *Pgk-1* sequences from polyploid accessions are grouped with those of the diploid parental species. The tree has two major clades: the one including sequences of the A genome and the second those derived from the genomes B, D, G and S. In Clade I, the A genome specific sequences from three *T. petropavlovskyi* accessions and from *Triticum* species (except *T. aestivum* cv. Chinese Spring) formed a group with 90% bootstrap value and 100% posterior probabilities support. One *T. petropavlovskyi* accession (AS358), together with two accessions of *T. polonicum* (AS304 and PI42209), formed a subclade, with bootstrap value of 95%. In Clade II, the B genome sequences from three *T. petropavlovskyi* accessions clustered together in a well supported (71% BS and 98% PP) subclade. SHW-DPW had a topology contiguous with two accessions of *T. polonicum* (AS302 and AS304), with 63% bootstrap support. The sequences from the D genomes mapped to two subclades. One consisted of three accessions of *T. petropavlovskyi*, eleven of *T. aestivum* and one of *Ae. tauschii* (TA1691), with 72% bootstrap support. The second one included SHW-DPW and *Ae. Tauschii* (AS60) with 87% BS and 100% PP.

**Figure 3:**
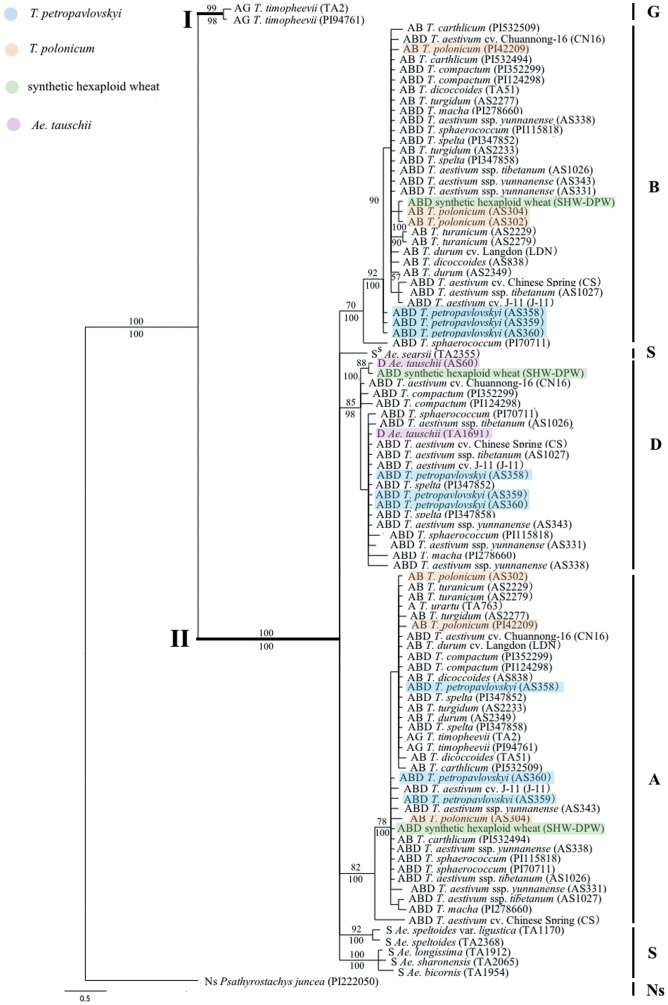
Bayesian tree inferred from the intron sequences of the *Pgk-1* gene of *T. petropavlovskyi* and its related species. Numbers above nodes are bootstrap values >50%; below nodes are Bayesian posterior probability values >90%. Genome composition, species name and accession number/cultivar name are indicated for each taxon.

ML analysis of the intron data yielded a single phylogenetic tree (−Lnlikelihood = 1804.87), with parameters: A = 0.29; C = 0.18; G = 0.17; T = 0.36 and gamma shape parameter = 0.60. The Bayesian analysis generated the same topology, as illustrated in Figure 3. Two major clades are evident: Clade I includes only the *Pgk-1* sequences from the G genome with a high bootstrap support (99% BS, 98% PP). The Clade II includes A, B, D and S genomes sequences and is congruent with the tree inferred from the exon+intron data, except for nodes presenting different statistical support. In Clade II, sequences from the B genome clustered together with a good support (70% BS and 100% PP). In this B genome clade, with the exception of *T. sphaerococcum* (PI70711), all accessions were grouped in a well supported subclade (92% BS and 100% PP); the three accessions of *T. petropavlovskyi* were separated from other *Triticum*. In the A genome subclade, *T. petropavlovskyi* (AS359) mapped together with *T. polonicum* (AS304) (78% BS and 100% PP).

**Figure 4:**
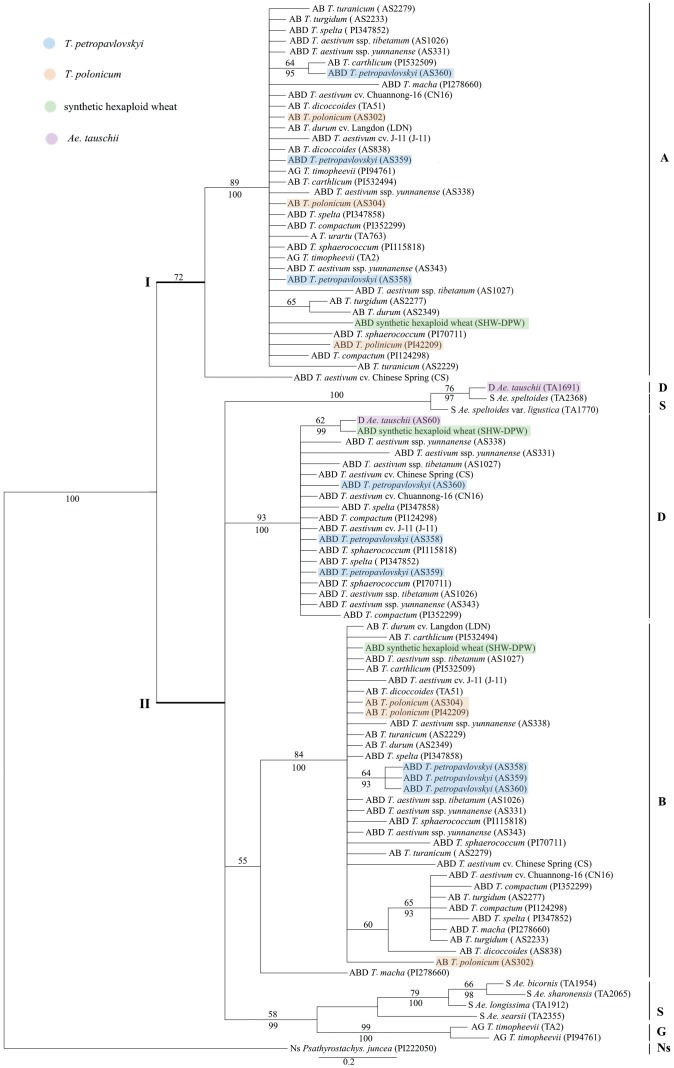
Maximum-likelihood tree inferred from the exon sequences of the *Pgk-1* gene of *T. petropavlovskyi* and its related species. Numbers above nodes are bootstrap values ≥50%; below nodes are Bayesian posterior probability values ≥90%. Genome composition, species name and accession number/cultivar name are indicated for each taxon.

Exon data generated a single ML phylogenetic tree (−Lnlikelihood = 2150.29), with the following parameters A = 0.24, C = 0.21, G = 0.27, T = 0.28, gamma shape parameter = 0.38. ML and BI analysis of the same data supported a similar topology. Figure 4 reports the ML tree of exon sequences which includes two major clades: Clade I, consisting of A genome sequences: *T. carthlicum* (PI532509) and *T. petropavlovskyi* (AS360) are mapped in the same group (64% BS and 95% PP). In the second clade (Clade II), which includes B, D, G, and S genomes, the B genome sequences of three accessions of *T. petropavlovskyi* grouped together (64% BS and 93% PP), separated from other accessions. *Ae. tauschii* (TA1691) and *Ae. speltoides* (TA2368) clustered in a subclade (76% BS and 97% PP).

### Network analysis

**Figure 5:**
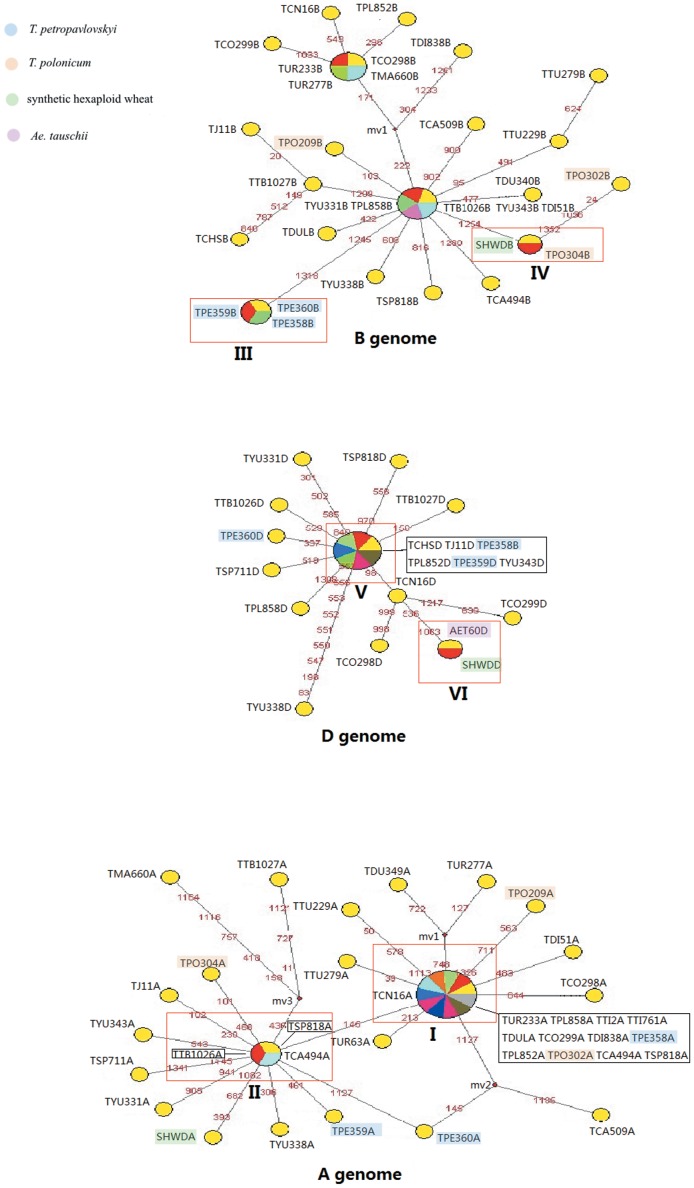
Median-joining (MJ) network inferred from the exon+intron sequences of the *Pgk-1* gene of *T. petropavlovskyi* and its related species. Abbreviations of the species names in the MJ network are listed in Table 1. Haplotypes in the network are represented by circles. Distance between nodes is proportional to the number of nucleotide substitutions among sequences.

**Figure 6:**
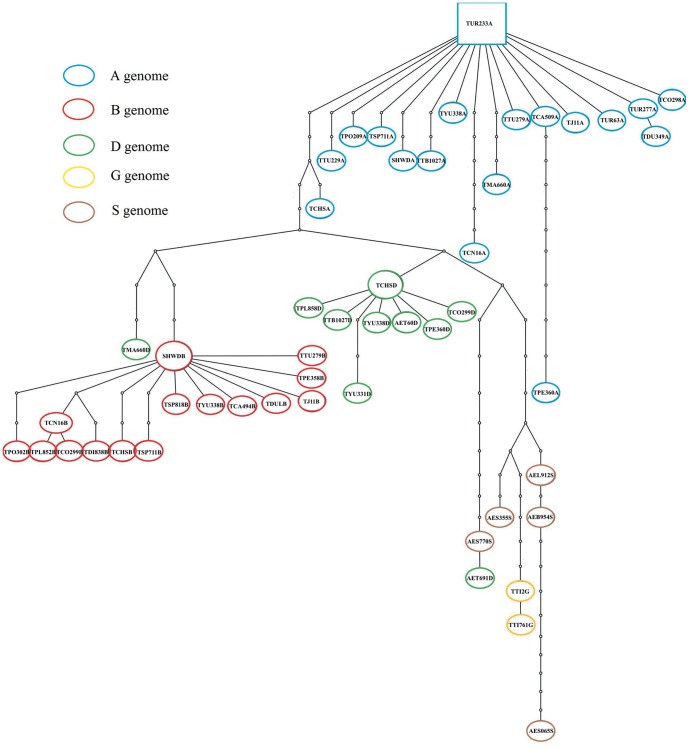
TCS network inferred from the exon sequences of the *Pgk-1* gene of *T. petropavlovskyi* and its related species. Abbreviations of species names are listed in Table 1. Haplotypes in the network are represented by circles of different color corresponding to the genomes indicated.

To highlight the relationships among haplotypes of the *Pgk-1* sequence, network methods were employed and the exon+intron data (Fig. 5) and exon data (Fig. 6) were considered. In Figure 5, each circular network node represents a haplotype, with node size being proportional to number of its isolates. Mv (median vectors representing missing intermediates) shows unsampled nodes inferred from the MJ network analysis. The number on the branches indicates the positions of the mutations. Network loops represent either true reticulation events or alternative genealogies in closely related lineages. MJ analysis of the *Pgk-1* exon+intron data recovered groupings corresponding to clades revealed by ML phylogeny. *T. petropavlovskyi*, as expected, was present in three clusters (A, B and D), representing the A, B and D genomes. Most accessions of *T. aestivum*, except *T. aestivum* cv. Chinese spring, were included in the A-type. The A-type sequences of three accessions of *T. petropavlovskyi* were included in subgroups I and II. *T. petropavlovskyi* (AS358) and *T. polonicum* (AS302) were placed at a central branching point. Meanwhile, in the B-type cluster, three accessions of *T. petropavlovskyi* grouped together in subgroup III, and *T. polonicum* (AS304) together with SHW-DPW in subgroup IV. In the D-type cluster, *T. petropavlovskyi* (AS358 and AS359), *T. aestivum* cv. Chinese Spring, *T. aestivum* cv. J-11, *T. aestivum* ssp. *yunnanense* (AS343) and *T. spelta* (PI347852) resulted included in subgroup V, while the sequences from the amphiploid SHW-DPW and *Ae. tauschii* formed the distinct subgroup VI. The TCS procedure [36] was used to illustrate haplotype relationships among accessions. TCS defined a 95% parsimony connection limit of 13 steps for exon alignment of fifty haplotypes derived from 96 sequences (Fig. 6). The TCS network consisted of three major haplotypic groups corresponding to the A, B and D genomes. The length of the branches between two nodes was proportional to the nucleotidic difference. In TCS analysis, *T. petropavlovskyi* (AS360) shows a close haplotype relationship with *T. carthlicum* from Xinjiang, China, supporting the exon results of the ML and BI analyses. Two further differences between the TCS and MJ were noted. Firstly, the haplotype of the D genome of *T. macha* (PI278660) appeared related to haplotypes of the B genome accessions. Secondly, *Ae. tauschii* (TA1691) showed a close haplotype relationship with the S genome of *Ae. speltoides* var. *ligustica*.

### Molecular dating

**Figure 7:**
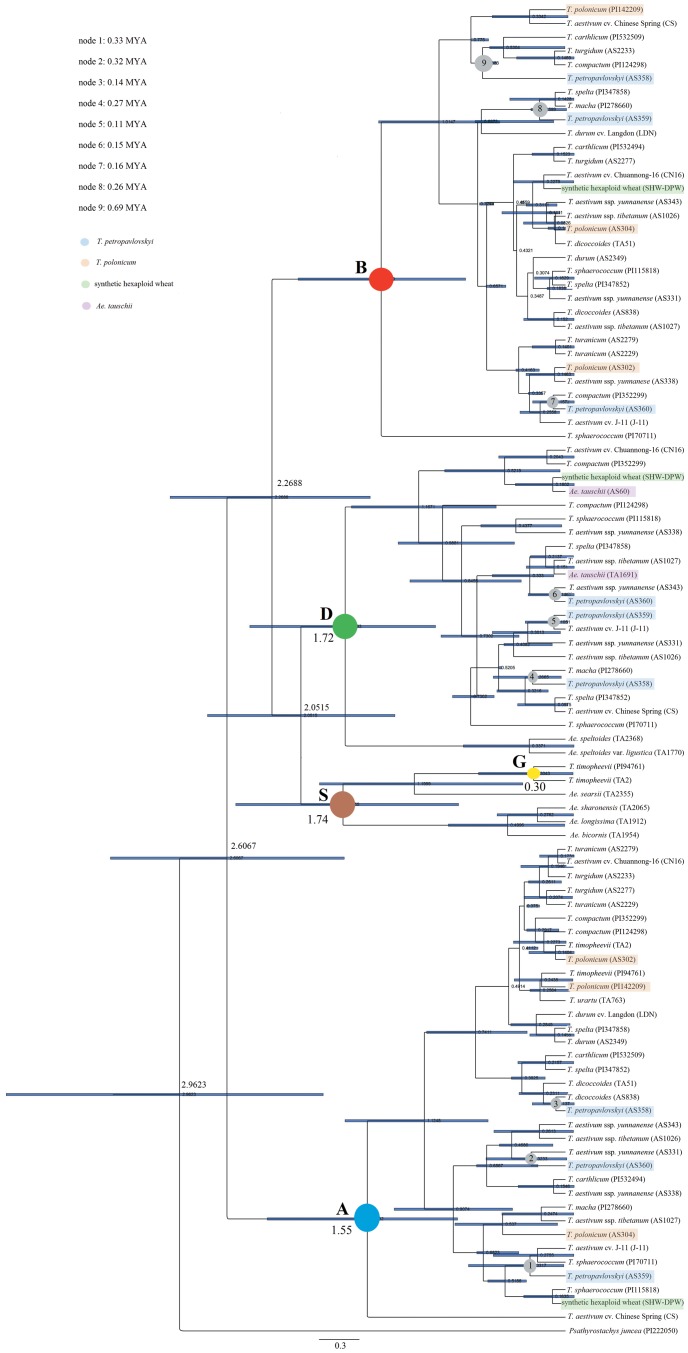
Time-calibrated tree based on intron region of the *Pgk-1* sequence of *T. petropavlovskyi* and related species using a Bayesian relaxed BEAST clock method. The different color of node labeled the genome information of the subclade. Numbers at nodes provide the estimated divergence dates.

The BEAST analysis of the intron region of *Pgk-1* was used to derive a time-calibrated phylogenetic tree (Fig. 7). Under a lognormal relaxed clock, rate variation was equal to 0.96 (95% C.I., 0.67–1.39), supporting the adoption of the relaxed clock method. The Yule prior was equal to 0.47 (95% C.I., 0.37–0.63) and five homoeologous types of the *Pgk-1* gene, A-, B-, D, G- and S-type, clustered in distinct clades. The divergence time of the A, B, and D genomes of *T. petropavlovskyi* was estimated equal to 1.13 (95% C.I., 0.65–1.75), 1.02 (95% C.I., 0.24–1.51), and 0.73 MYA (95% C.I., 0.41–1.01), respectively. The split between *Pgk-1* A and D genomes of *T. petropavlovskyi* and its putative diploid genome donor, *T. polonicum* and *Ae. tauschii*, took place around 0.74–1.13 and 0.33–0.73 MYA, respectively. The B genome diverged from the S genome at 2.27 MYA (95% C.I., 1.68–3.19), while the divergence time of *T. petropavlovskyi* and *T. polonicum* was 0.68–0.91 MYA for A genome. The divergence time of *T. petropavlovskyi* and *T. polonicum* and the B genome was 0.34–0.78 MYA. The divergence time of *T. petropavlovskyi* from hexaploid wheat resulted equal to 0.14–0.33 MYA (A genome), 0.16–0.69 MYA (B genome) and 0.11–0.27 MYA (D genome), respectively (node 1–node 9). In the tree, the tetraploid wheat *T. polonicum* diverged earlier than *T. petropavlovskyi*. In the A genome, the divergence time of *T. petropavlovskyi* (AS358) was later than the other two accessions of *T. petropavlovskyi*. On the contrary, in B and D genomes, the divergence time was earlier than the other two accessions. Additionally, the divergence time of B genome of *T. petropavlovskyi* was earlier than other three Chinese endemic wheat landraces. Between *T. petropavlovskyi* and SHW-DPW, a significant difference was observed: in the genomes A, B and D, the divergence time of SHW-DPW was later than *T. petropavlovskyi*.

## Discussion

### Relationships between *T. petropavlovskyi* and hexaploid wheat taxa

Based on cytological results, Yao et al. [3] and Chen et al. [23] suggested that the B genome was responsible for the difference between *T. petropavlovskyi* and *T. aestivum* cv. Chinese Spring, and that two pairs of chromosomes, one identified as chromosome 6B [2], were involved. The allelic variation at the HMW glutenin subunits loci, *Gli-1* and *Gli-2*, supported the cytological results [59]. Also, Yang et al. [10] reported that *T. petropavlovskyi* differed from *T. spelta* in at least one or two pairs of chromosomes. Results based on molecular markers, including A-PAGE, SDS-PAGE, STS-PAGE, SSR and RFLP, indicate that *T. petropavlovskyi* is genetically distinct from other Chinese endemic wheat landraces [5], [59].

Our ML and BI study of the *Pgk-1* gene indicates that the A and D genomes of *T. petropavlovskyi* are basically shared with *T. spelta*, *T. compactum* and *T. sphaerococcum*. When the B genome is considered, *T. petropavlovskyi* groups in one subclade, comparatively distantly related to *T. aestivum*. In addition, the B-type of MJ network shows that the accessions of *T. petropavlovskyi* (subgroup III) are distinct from those of other species. SHW-DPW, a synthetic hexaploid wheat with both genomes of *T. polonicum* and *Ae. tauschii* [26], based on the *Pgk-1* gene is characterized by the A, B and D genomes, the SHW-DPW is distant from those of *T. petropavlovskyi* in B and D genomes.

### Relationships between *T. petropavlovskyi* and the tetraploid wheats

Morphologically, the spikelet of *T. petropavlovskyi* are similar to those of *T. turanicum* and *T. polonicum* [7], [26]. Moreover, the cytology of interspecific hybrids between *T. petropavlovskyi* and tetraploid wheats support a closer relationship with the AABB genomes, compared to wheats with the AAGG genome [2]. According to Akond and Watanabe [24], *T. petropavlovskyi* is more closely related to *T. polonicum* than to *T. durum* or *T. turgidum*. However, Arbuzova et al. [60] and Efremova et al. [18] report that the genes supporting the elongated glumes in *T. polonicum* and *T. petropavlovskyi* are not allelic.

In the present study, based on the ML and BI analyses of the genome A *Pgk-1* gene, two accessions of *T. polonicum* have a common topology with *T. petropavlovskyi*, while the TCS analysis of exon data indicates that the haplotype of A genome in *T. petropavlovskyi* (AS360) is more closely related to *T. carthilicum* than to other wheats. The phylogenetic analyses and MJ network specific for the B genome shows that three accessions of *T. petropavlovskyi* group together, and are topologically distant from those of tetraploid wheats. This finding indicates that the B genome of *T. petropavlovskyi* diverge from the one of tetraploid species. Yao et al. [3] and Chen et al. [23] also recognized cytologically that the B genome of *T. petropavlovskyi* was different from those of hexaploid wheats.

### Relationships between *T. petropavlovskyi* and *Ae. tauschii*

Based on RFLPs, Ward et al. [5] found that *T. petropavlovskyi* is genetically more closely related to accessions of *Ae. tauschii* from Iran than from China. Yang et al. [10] concluded that *T. petropavlovskyi* is derived from a hybrid between *Ae. tauschii* and a presumed *T. polonicum* genotype. However, the phylogenetic analysis of the *Acc-1* genes indicates that the D genome of *T. petropavlovskyi* is very similar to the D genome orthologs of *T. aestivum* and only distantly related to *Ae. tauschii* [26]. In the present study, D genomes of *Ae. tauschii* belong to two different clusters. One groups with SHW-DPW, *T. compactum* and *T. aestivum* cv. Chuannong-16, while *T. petropavlovskyi*, *T. macha*, *T. spelta*, *T. sphaerococcum* and three Chinese endemic wheats are included in a clade with *Ae. tauschii* (TA1691). Based on TCS analysis, *T. petropavlovskyi* shares common topologies with the hexaploid species D genomes. The sequence of the *Pgk-1* gene from TA1691 is significantly different from those of other accessions of *Ae. tauschii*, in agreement with Huang et al. [31]. Together, available results supports that the D genome of *T. petropavlovskyi* is similar to D genome orthologs of *T. aestivum* and only distantly related to *Ae. tauschii*.

### The divergence time of the *T. petropavlovskyi*

Huang et al. [31] report that the diploid progenitors of the A, B and D genomes present in diploid, tetraploid, and hexaploid wheats radiated between 2.5 and 4.5 MYA. We report that the divergence time of the A, B and D genomes corresponds to 2.61 (95% C.I., 1.77–3.55), 2.27 (95% C.I., 1.69–3.19) and 2.05 MYA (95% C.I., 1.40–2.81), respectively. The divergence of the B genome of *T. petropavlovskyi* from those of other wheats is dated in this paper is from 0.16 (95% C.I., 0–0.38) to 0.69 MYA (95% C.I., 0.55–0.70), the earliest date for the four Chinese endemic wheat landraces we considered.

Concerning the A and D genome of *T. petropavlovskyi*, the resulting divergence times are 0.14–0.33 and 0.11–0.27 MYA, respectively, values similar to those of other hexaploid species. The divergence time of A genome of *T. petropavlovskyi* from *T. polonicum* varies from 0.68 (95% C.I., 0.41–0.71) to 0.90 MYA (95% C.I., 0.45–1.41), a divergence earlier than those between *T. petropavlovskyi* and hexaploid species. The divergence time results indicate that *T. polonicum* may have played a role in the evolutionary history of *T. petropavlovskyi*.

### The possible origin of *T. petropavlovskyi*

 This study shows that the *Pgk-1* sequences of the A genome of *T. petropavlovskyi* group with *T. polonicum*. For the *Pgk-1* locus of the D genome, the accessions of *Ae. tauschii* just cluster with the amphiploid SHW-DPW. The *Pgk-1* B genome data indicate that *T. petropavlovskyi* is distantly related to the other three Chinese endemic wheat landraces. The MJ network results are congruent with the results reported above. Also the TCS analysis supports the conclusion that the relationship among haplotypes of *T. petropavlovskyi* and *T. polonicum* have very similar A and B genomes. We report a distant relationship between *T. petropavlovskyi* and *Ae. tauschii*. We conclude that *T. petropavlovskyi* is neither derived from an independent allopolyploidization event nor from a single mutation in *T. aestivum*. It is most likely that *T. petropavlovskyi* has an origin starting with a natural cross between *T. aestivum* and *T. polonicum*, with that event taking place around 0.78 MYA.

## References

[1] Yen C, Yang JL, Luo MC (1988) The origin of the Tibetan weedrace of hexaploid wheat, Chinese Spring, Chengdu guangtou and other landraces of white wheat complex from china. In Proceedings of the 7^th^ International Wheat Genetics Symposium Miller TE, Koebner RMD, eds. Cambridge, UK. pp 175–179.

[2] Chen Q, Sun YZ, Dong YS (1985) Cytogenetical studies on interspecific hybrids of Xinjiang wheat. Acta Agron Sin 11: 23–28.

[3] Yao JX (1983) Research on a new species in *Triticum*-Xinjiang wheat with rice-like spike. Hereditas (Beijing) 5: 17–20.

[4] Kim HS, Ward RW (2000) Pattern of RFLP-based genetic diversity in germplasm pools of common wheat with different geographical or breeding program origins. Euphytica 115: 197–208.

[5] Ward RW, Yang ZL, Kim HS, Yen C (1998) Comparative analyses of RFLP diversity in landraces of *Triticum aestivum* and collections of *Aegilops tauschii* from China and South Asia. Theor Appl Genet 96: 312–318.

[6] Jakubtsiner MM (1959) K poznaniyu pshenits Kitaja/A contirbution to the knowledge of the wheats of China. Bot J 44: 1425–1436.

[7] Udaczin RA, Miguschova EF (1970) Novoe v poznanii roda *Triticum* L.. Venstnik S-Kh Nauki 9: 20–24.

[8] Riley R, Coucoli H, Chapman V (1967) Chromosomal interchanges and the phylogeny of wheat. Heredity 22: 233–247.

[9] Shao QQ, Li CS, Basang CR (1980) Semi-wild wheat from Xizang (Tibet). Acta Genet Sin 7: 150–156.

[10] Yang WY, Yen C, Yang JL (1992) Cytogenetic study on the origin of some special Chinese landraces of common wheat. Wheat Inform Serv 75: 14–20.

[11] Dorofeev VF, Filatenko AA, Migushova EF, Udaczinz RA, Jakubziner MM (1979) Flora of Cultivated plants. In Wheat Dorofeev VF, Migushova EKolos, L., eds. pp 1–384.

[12] Watanabe N, Imamura I (2002) The inheritance and chromosomal location of a gene for long glume phenotype in *Triticum petropavlovskyi* Udacz. et Migusch. J Genet Breed 57: 221–227.

[13] Watanabe N, Sekiya T, Sugiyama K, Yamagishi Y, Imamura I (2002) Telosomic mapping of the homoeologous genes for the long glume phenotype in tetraploid wheat. Euphytica 128: 129–134.

[14] Wang HY, Huang XQ, Röder MS, Börner A (2002) Genetic mapping of loci determining long glumes in the genus *Triticum* . Euphytica 123: 287–293.

[15] Yen C, Yang JL, Liu XD, Li LR (1983) The distribution of *Aegilops tauschii* Cosson in China with reference of the origin of the Chinese common wheat. In Proceedings of the 7^th^ International Wheat Genetics Symposium, Sakamoto S, eds. Kyoto, Japan. pp 55–58.

[16] Yang RW, Zhou YH, Zheng YL (2001) Analysis on chromosome C-banding of dwarf polish wheat (*Triticum polonicum*). J Sichuan Agric Univ 19: 112–114.

[17] Liu GX, Zhou YH, Zheng YL, Yang RW, Ding CB (2002) Morphological and cytological studies of dwarfing polish wheat (*Triticum turgidum* concv. *polonicum*) from Xinjiang, China. J Sichuan Agric Univ 20: 189–193.

[18] Efremova TT, Maystrenko OI, Laikova LI, Arbuzova VS, Popova OM (2000) Comparative genetic analysis of hexaploid wheats *Triticum petropavlovskyi* Udasz. et Migusch. and *Triticum aestivum* L. Russ J Genet 36: 1142–1148. 11094748

[19] Yang RW, Zhou YH, Zheng YL, Hu C (2000) Genetic differences and the relationship of gliadin between Triticum polonicum and Triticum petropavlovskyi. J Triticeae Crops 20: 1–5.

[20] Chen QF (1999) Discussion on origin of Chinese endemic wheat. Guizhou Agri Sci 27: 20–25.

[21] Goncharov NP (2005) Comparative genetic analysis-a base for wheat taxonomy revision. Czech J Genet Plant Breed 41: 52–55.

[22] Akond ASMGM, Watanabe N, Furuta Y (2008) Comparative genetic diversity of *Triticum aestivum-Triticum polonicum* introgression lines with long glume and *Triticum petropavlovskyi* by AFLP-based assessment. Genet Resour Crop Evol 55: 133–141.

[23] Chen PD, Liu DJ, Pei GZ, Qi LL, Huang L (1988) The chromosome constitution of three endemic hexaploid wheats in western China. In Proceedings of the 7^th^. International Wheat Genetics Symposium Miller TE, Koebner RMD, eds. Cambridge, UK. pp 75–80.

[24] Akond ASMGM, Watanabe N (2005) Genetic variation among Portuguese landraces of ‘Arrancada’ wheat and *Triticum petropavlovskyi* by AFLP-based assessment. Genet Resour Crop Evol 52: 619–628.

[25] Kang HY, Wang Y, Yuan HJ, Jiang Y, Zhou YH (2008) A new synthesized 6x-wheats, derived from dwarfing polish wheat (*Triticum polonicum* L.) and *Aegilops tauschii* Cosson. Intern J Agric Res 3: 252–260.

[26] Kang HY, Fan X, Zhang HQ, Sha LN, Sun GL, et al (2010) The origin of *Triticum petropavlovskyi* Udacz. et Migusch.: demonstration of the utility of the genes encoding plastid acetyl-CoA carboxylase sequence. Mol Breed 25: 381–395.

[27] Sang T (2002) Utility of low-copy nuclear gene sequences in plant phylogenetics. Crit Rev Biochem Mol Biol 37: 121–147. 12139440 10.1080/10409230290771474

[28] Smith J, Funke M, Woo V (2006) A duplication of gcyc predates divergence within tribe Coronanthereae (Gesneriaceae): phylogenetic analysis and evolution. Plant Syst Evol 261: 245–256.

[29] Chalupska D, Lee HY, Faris JD, Evrard A, Chalhoub B, et al (2008) *Acc* homoeoloci and the evolution of wheat genomes. Proc Natl Acad Sci 105: 9691–9696. 18599450 10.1073/pnas.0803981105 PMC2474508

[30] Huang SX, Sirikhachornkit A, Faris JD, Su XJ, Gill BS, et al (2002) Phylogenetic analysis of the acetyl-CoA carboxylase and 3-phosphoglycerate kinase loci in wheat and other grasses. Plant Mol Biol 48: 805–820. 11999851 10.1023/a:1014868320552

[31] Huang SX, Sirikhachornkit A, Su XJ, Fairs J, Gill BS, et al (2002) Genes encoding plastid acetyl-CoA carboxylase and 3-phosphoglycerate kinase of the *Triticum/Aegilops* complex and the evolutionary history of polyploid wheat. Proc Natl Acad Sci 99: 8133–8138. 12060759 10.1073/pnas.072223799 PMC123033

[32] Golovnina KA, Glushkov SA, Blinov AG, Mayorov VI, Adkison LR, et al (2007) Molecular phylogeny of the genus *Triticum* . Plant Syst Evol 264: 195–216.

[33] Yan C, Sun GL, Sun DF (2011) Distinct origin of the Y and St genome in *Elymus* species: evidence from the analysis of a large sample of St genome species using two nuclear genes. PLoS ONE 6: e26853. 22046383 10.1371/journal.pone.0026853 PMC3203181

[34] Wu F, Mueller LA, Crouzillat D, Pétiard V, Tanksley SD (2006) Combining bioinformatics and phylogenetics to identify large sets of single-copy orthologous genes (COSII) for comparative, evolutionary and systematic studies: a test case in the Euasterid plant clade. Genetics 174: 1407–1420. 16951058 10.1534/genetics.106.062455 PMC1667096

[35] Sun GL, Ni Y, Daley T (2008) Molecular phylogeny of *RPB2* gene reveals multiple origin, geographic differentiation of H genome, and the relationship of the Y genome to other genomes in *Elymus* species. Mol Phylogenet Evol 46: 897–907. 18262439 10.1016/j.ympev.2007.12.024

[36] Sun GL, Salomon B (2009) Molecular evolution and origin of tetraploid *Elymus* species. Breed Sci 59: 487–491.

[37] Petersen G, Seberg O, Yde M, Berthelsen K (2006) Phylogenetic relationships of *Triticum* and *Aegilops* and evidence for the origin of the A, B, and D genomes of common wheat (*Triticum aestivum*). Mol Phylogenet Evol 39: 70–82. 16504543 10.1016/j.ympev.2006.01.023

[38] Fan X, Sha LN, Zeng J, Kang HY, Zhang HQ, et al (2012) Evolutionary dynamics of the *Pgk-1* gene in the polyploid genus *Kengyilia* (Triticeae: Poaceae) and its diploid relatives. PLoS ONE 7: e31122. 22363562 10.1371/journal.pone.0031122 PMC3282717

[39] Gornicki P, Faris J, King I, Podkowinski J, Gill BS, et al (1997) Plastid-localized acetyl-CoA carboxylase of bread wheat is encoded by a single gene on each of the three ancestral chromosome sets. Poc Natl Acad Sci 94: 14179–14184. 10.1073/pnas.94.25.14179 PMC28453 9391173

[40] Kilian B, Özkan H, Deusch O, Effgen S, Brandolini A, et al (2007) Independent wheat B and G genome origins in outcrossing *Aegilops* progenitor haplotypes. Mol Biol Evol 24: 217–227. 17053048 10.1093/molbev/msl151

[41] Doyle JJ, Doyle JL (1990) Isolation of plant DNA from fresh tissue. Focus 12: 13–15.

[42] Doyle JJ, Doyle JL (1999) Nuclear protein-coding genes in phylogeny reconstruction and homology assessment: some examples from Leguminosae. In The Molecular Systematics and Plant Evolution Hollingsworth PM, Bateman RM, Gornall RJ, eds. Taylor and Francis, London. pp 229–254.

[43] Fan X, Sha LN, Yang RW, Zhang HQ, Kang HY, et al (2009) Phylogeny and evolutionary history of *Leymus* (Triticeae; Poaceae) based on a single-copy nuclear gene encoding plastid acetyl-CoA carboxylase. BMC Evol Biol 9: 247. 19814813 10.1186/1471-2148-9-247 PMC2770499

[44] Thompson JD, Plewniak F, Poch O (1999) A comprehensive comparison of multiple sequence alignment programs. Nucleic Acids Res 27: 2682–2690. 10373585 10.1093/nar/27.13.2682 PMC148477

[45] Tamura K, Peterson D, Peterson N, Stecher G, Nei M, et al (2011) MEGA5: molecular evolutionary genetics analysis using maximum likelihood, evolutionary distance, and maximum parsimony methods. Mol Biol Evol 28: 2731–2739. 21546353 10.1093/molbev/msr121 PMC3203626

[46] Posada D, Crandall KA (1998) Modeltest: testing the model of DNA substitution. Bioinformatics 14: 817–818. 9918953 10.1093/bioinformatics/14.9.817

[47] Felsenstein J (1985) Confidence limits on phylogenies: an approach using the bootstrap. Evolution 39: 783–791. 28561359 10.1111/j.1558-5646.1985.tb00420.x

[48] Huelsenbeck JP, Ronquist F (2001) MrBayes: Bayesian inference of phylogenetic trees. Bioinformatics 17: 754–755. 11524383 10.1093/bioinformatics/17.8.754

[49] Allaby RG, Brown TA (2001) Network analysis provides insights into evolution of 5S rDNA arrays in *Triticum* and *Aegilops* . Genetics 157: 1331–1341. 11238418 10.1093/genetics/157.3.1331 PMC1461568

[50] Wang HY, Wang XE, Chen PD, Liu DJ (2007) Assessment of genetic diversity of Yunnan, Tibetan, and Xinjiang wheat using SSR markers. J Genet Genomics 34: 623–633. 17643948 10.1016/S1673-8527(07)60071-X

[51] Bordbar F, Rahiminejad MR, Saeidi H, Blattner FR (2011) Phylogeny and genetic diversity of D-genome species of *Aegilops* and *Triticum* (Triticeae, Poaceae) from Iran based on microsatellites, ITS, and *trn*L-F. *Plant* . Syst Evol 291: 117–131.

[52] Yan C, Sun GL (2011) Nucleotide divergence and genetic relationships of *Pseudoroegneria* species. Biochem Syst Ecol 39: 309–319.

[53] Huson DH, Bryant D (2006) Application of phylogenetic networks in evolutionary studies. Mol Biol Evol 23: 254–267. 16221896 10.1093/molbev/msj030

[54] Clement M, Posada D, Crandall KA (2000) TCS: a computer program to estimate gene genealogies. Mol Ecol 9: 1657–1659. 11050560 10.1046/j.1365-294x.2000.01020.x

[55] Drummond AJ, Suchard MA, Xie D, Rambaut A (2012) Bayesian phylogenetics with BEAUti and the BEAST 1.7. Mol Biol Evol 29: 1969–1973. 22367748 10.1093/molbev/mss075 PMC3408070

[56] Gaut BS (1998) Molecular clocks and nucleotide substitution rates in higher plants. In The Evolutionary Biology Hecht MK, ed. Plenum Press New York, .pp 93–120.

[57] Wolfe KH, Gouy M, Yang YW, Sharp PM, Li WH (1989) Date of the monocot-dicot divergence estimated from chloroplast DNA sequence data. Proc Natl Acad Sci 86: 6201–6205. 2762323 10.1073/pnas.86.16.6201 PMC297805

[58] Wolfe KH, Li WH, Sharp PM (1987) Rates of nucleotide substitution vary greatly among plant mitochondrial, chloroplast, and nuclear DNAs. Proc Natl Acad Sci 84: 9054–9058. 3480529 10.1073/pnas.84.24.9054 PMC299690

[59] Wei YM, Zheng YL, Liu DC, Zhou YH, Lan XJ (2002) HMW-glutenin and gliadin variations in Tibetan weedrace, Xinjiang rice wheat and Yunnan hulled wheat. Genet Resour Crop Evol 49: 327–330.

[60] Arbuzova V S, Efremova TT, Laikova LI, Maystrenko OI, Popova OM, et al (1996) The development of precise genetic stocks in two wheat cultivars and their use in genetic analysis. Euphytica 89: 11–15.

