# Correction: Evolutionary History of *Triticum petropavlovskyi* Udacz. et Migusch. Inferred from the Sequences of the 3-Phosphoglycerate Kinase Gene

**DOI:** 10.1371/annotation/51159241-2192-46c3-a4f1-a09f590e1f08

**Published:** 2014-01-03

**Authors:** Qian Chen, Hou-Yang Kang, Xing Fan, Yi Wang, Li-Na Sha, Hai-Qin Zhang, Mei-Yu Zhong, Li-Li Xu, Jian Zeng, Rui-Wu Yang, Li Zhang, Chun-Bang Ding, Yong-Hong Zhou

The last sentence of the Abstract is incorrect. Please see the corrected sentence here:

"The dating of divergence among *T. polonicum, T. petropavlovskyi, T. carthlicum, T. turgidum,* and *T. compactum* indicates an age of 0.78 million years."

The last sentence of the Discussion is incorrect. Please see the corrected sentence here:

"It is most likely that *T. petropavlovskyi* has an origin starting with a natural cross between *T. aestivum* and *T. polonicum*."

